# Knowledge, attitudes, and practice around urinary tract infections of general practice assistants in the Netherlands: a cross-sectional internet survey

**DOI:** 10.1186/s12875-025-03025-3

**Published:** 2025-11-03

**Authors:** Stefan M.L. Cox, Luka T.F. Nicolaes, Tamara N. Platteel, Jochen W.L. Cals, Eefje G.P.M. de Bont

**Affiliations:** 1 Department of Family Medicine, CAPHRI Care and Public Health Institute, Maastricht University, Maastricht, Netherlands; 2 Julius Center for Health Sciences and Primary Care, University Medical Center Utrecht, Utrecht, Netherlands

**Keywords:** Urinary tract infections, Cystitis, General practice, General practice assistants, Antibiotic stewardship, Diagnostics

## Abstract

**Background:**

In Dutch general practice, urinary tract infections (UTIs) are the most common indication for prescribing antibiotics. General practice assistants (GPAs) are the first point of contact for patients with UTI-associated symptoms and sometimes even manage these cases without consulting a general practitioner. Nevertheless, literature on how GPAs provide and experience UTI-care is limited.

**Methods:**

To investigate the knowledge, attitude, and practice of Dutch GPAs regarding UTIs in general practice, we constructed a cross-sectional online survey. The survey assessed actively working Dutch GPAs’ knowledge, practice, and attitude in UTI-care. Participants were recruited through social media platforms in May and June 2024. Descriptive statistics were used to perform primary data-analysis. Secondary analysis was performed using univariate and multivariate logistic regression models.

**Results:**

478 of the 643 obtained responses were eligible for analysis. Results showed 95.8% of the GPAs think their UTI knowledge is sufficient. However, only one-fourth of respondents selected all correct groups at higher risk of developing a complicated UTI. Additionally, almost 70% of the respondents would perform urinalysis as a precaution if a patient hands in urine, even when UTI-associated symptoms are absent. Nine out of ten GPAs would *never* disregard urinalysis results. Furthermore, while GPAs indicated to apply shared decision-making often, wait-and-see policies are not regularly advised.

**Conclusions:**

GPAs seem to be unaware of their limitations regarding UTI-care, especially overvaluing the urine dipstick as a diagnostic tool. GPAs should adjust their preconceived notions of patient preferences, since patients’ willingness to try non-antibiotic treatments is higher than they think.

**Supplementary Information:**

The online version contains supplementary material available at 10.1186/s12875-025-03025-3.

## Background

Urinary tract infections (UTIs) are the most common bacterial infections seen in general practice. In 2022, around 149 out of 1000 inhabitants of the Netherlands were diagnosed with a cystitis by their general practitioner (GP), which accounts for 2.6% of all GP consultations [1]. In general practice, the lifetime prevalence of uncomplicated UTI for women is 40–60%, and in most cases the first episode takes place before the age of 25 years [2–5].

The primary diagnostic tool used in general practice in the Netherlands is the urinary dipstick test, which indicates whether i.a. nitrite and/or leukocytes are present. According to Dutch guidelines, the point-of-care diagnosis of UTIs should be based on UTI-related symptoms in combination with a positive nitrite result. In the event of a negative nitrite test paired with a positive leukocyte test, the test is inconclusive and additional diagnostic tests should be considered. Despite its suboptimal specificity (66%) and sensitivity (75%), the urine dipstick plays an important role in the diagnostic algorithm of UTIs [6, 7].

In the Netherlands, general practice assistants (GPAs) are the primary point of contact for patients with potential UTI-related symptoms. GPAs perform (telephone) triage, medical and diagnostic procedures, administrative tasks, and advise and inform patients [8]. GPAs frequently manage UTI cases independently; therefore they play a crucial role in diagnosing and treating UTIs in GP’s offices. Previous research revealed that GPs regularly act outside of established guidelines when diagnosing UTIs [9, 10]. However, studies on how UTI-care is provided and experienced by GPAs in general practice are limited. Therefore, the aim of this study is to investigate knowledge, attitude, and practice of Dutch GPAs towards UTIs in general practice.

## Methods

### Study design

To elucidate the knowledge, attitudes, and practice around UTIs of GPAs in the Netherlands we conducted a cross-sectional internet-based survey between the third week of May and first week of June 2024 in the Netherlands. The digital survey was designed using Qualtrics software (Qualtrics, Provo, Utah). Participants were recruited through posts on several social media platforms including LinkedIn, Facebook, WhatsApp, Telegram, and Instagram. The announcements with the survey link were posted on general pages and Dutch group pages specifically created for GPAs. We also spread the link through regional care networks on infection prevention and antimicrobial resistance (IP & AMR) [11]. After two weeks reminders were sent and posted.

### Setting and subjects

Respondents actively working as a GPA in Dutch GP’s offices were eligible for participation, as well as GPAs in training. Dutch GPAs training, tasks, and continuing education are explained in Table 1. We excluded respondents that exclusively worked at out-of-hours centres. Based on an estimated population of 35.000 GPAs in the Netherlands, a confidence interval of 95%, a margin of error of 10% and a standard deviation of 0.5, a sample size of 96 was considered to be adequate to ensure generalisability (12).

**Table 1:** Training, tasks, and continuing education of Dutch GPAs

Training: BTEC Level 3/EQF-4 level with courses in pharmacology, anatomy and pathology, social and communicative skills, triage, medical technical procedures, good clinical practice, and practice management. Tasks: Triage, informing patients, administrative tasks, sample collection, performing (simple) diagnostic tests, wound care, and assisting with medical procedures. Continuing education: Limited and varies per region. GPAs must be in possession of a valid CPR certificate. GPs have to ensure that GPAs are competent and qualified. There is no mandatory quality registration for GPAs.	

### Data collection and content of the survey

The content of the survey was based on previous qualitative and quantitative studies, as well as input from experts on UTIs in general care [10, 12]. The survey was constructed specifically for this study and its contents can be viewed in Supplementary File 1. Face validity checks to optimize the quality and readability of the survey were performed by three GPAs, four GPs, four medical students, one PhD-candidate on UTIs, and three research assistants of the Department of Family Medicine of Maastricht University. All three GPAs were included from conception of the survey to ensure readability for the target population. Inclusion of the GPs and GPAs during survey validation also ensured content validity.

The survey consisted of 32 questions, varying between Likert-scale questions with several sub-items, true-false-do not know questions, yes-no questions, listing priorities- and regular multiple-choice questions. Questions that assessed the knowledge, attitude, and practice of GPAs on UTI-care were followed by questions to gather sociodemographic information. Open-ended questions on potential areas for improvement in the management of UTIs, and the possibility to offer comments on the survey, if any, were also included.

To enhance data quality, participants had to complete the current question before proceeding to the next question. To stimulate completion of the questionnaire, open ended questions did not require an answer to proceed with the survey [13, 14]. Respondents were not able to update their responses on prior questions. Respondents had one week to finish the survey upon starting. To prevent Ballot Box Stuffing, Qualtrics placed a cookie on participants’ browsers when a response was submitted, if not disagreed to by the participant.

### Data analysis

Responses on the survey were automatically transferred to SPSS Statistics Version 28.0.1.1. (IBM 2021, Armonk, New York). Descriptive statistics were used to analyse sociodemographic data. To describe population characteristics we calculated means, medians, standard deviations, ranges, and frequencies for pre-selected variables. Also, primary analysis on the questions concerning GPAs’ knowledge, attitude and practice on UTI-care was performed by using descriptive statistics, expressed in frequencies and cross-tabulations. The answers to open-ended questions were categorized. Unanswered questions were defined as missing data.

Secondary analysis on the potential influence of sociodemographic characteristics on the primary outcome measures was performed using χ^2^- or Fisher’s exact test for independent and dependent categorical variables. To identify independent categorical variables associated with dependent variables, Kruskal-Wallis H tests or Mann-Whitney U tests were performed, where applicable. Furthermore, univariate and multivariate regression models were used to calculate associations, using odds ratios (OR) and 95%-confidence intervals (CI). Independent variables used were age in years, working experience in years, number of working days per week, own experience with UTI, and own experience with antibiotics and UTI.

## Results

### Characteristics of study population

**Fig. 1:**
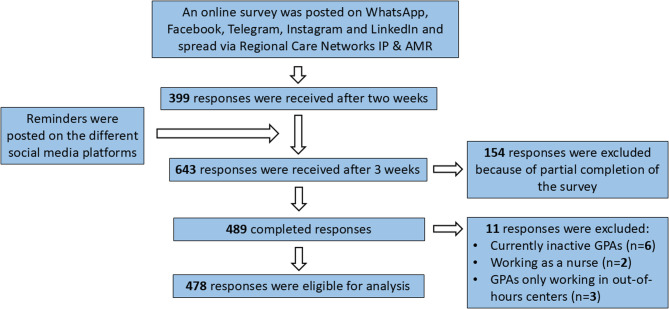
Flowchart of included responses*.GPA= general practice assistant; IP & AMR = infection prevention and antimicrobial resistance*

A total of 643 responses were received after three weeks. After exclusion of incomplete responses (*n* = 154) and responses from respondents who did not meet the inclusion criteria (*n* = 11), 478 responses remained eligible for analysis (Fig. 1).

Table 2 summarizes the characteristics of the participating GPAs. Participants had a mean age of 39.9 years (SD 11.6), and a median working experience of 10 years (range 0–43). The study population consisted of 476 women (99.6%) and 26 GPAs in training (5.4%). Almost a quarter of the respondents were active in the province of Limburg.

**Table 2:** Characteristics of the study population

Characteristics	GPAs (*n* = 478)
Female, n (%)	476 (99.6)
GPA in training, n (%)	26 (5.4)
Age, mean years (SD)	39.9 (11.6)
Years of profession, median (range)	10 (0–43)
Workweek, median days per week (range)	4.0 (1–5)
Medical history of UTI(s), n (%)	361 (75.5)
Obtained AB for a UTI in the past, n (%)	326 (68.2)
Working region, n (%)	
Limburg	117 (24.5)
Zuid-Holland	87 (18.2)
Gelderland	54 (11.3)
Brabant	52 (10.9)
Other 8 provinces	168 (35.1)

*GPA general practice assistant*,* SD tandard deviation*,* UTI urinary tract infection*,* AB antibiotics.*

### GPAs’ knowledge on UTIs

#### Perceived knowledge

GPAs’ knowledge on UTIs was assessed by multiple-choice questions asking for symptoms most indicative of a UTI and for patient groups that have a higher risk of developing a complicated UTI. In addition, 5 true-false questions on different aspects of UTI-care, were presented. Among the GPAs, 171 respondents (35.8%) *slightly agreed* and 287 respondents (60.0%) *strongly agreed* on the statement ‘*I am capable of providing a patient comprehensive and accurate information about UTIs*’. The degree of agreement with this statement was positively associated with working experience in a multivariate ordinal regression model (OR = 1.04; 95%CI = 1.01–1.07) (Supplementary Table 1).

Moreover, 92.7% of the GPAs slightly or strongly agreed on the statement ‘*My knowledge is sufficient to assess whether a patient has a UTI*,* based on their symptoms and dipstick analysis’*. GPA age was negatively associated with agreement to this statement (OR = 0.96, 95%CI = 0.94–0.99), while their working experience (OR = 1.03, 95%CI = 1.01–1.06) and they themselves having had an antibiotically treated UTI (OR = 3.31, 95%CI = 1.43–7.68) were positively associated in an ordinal regression model (Table 3).

**Table 3:** Ordinal regression results for the agreement of GPAs with the statement ‘my knowledge is sufficient to assess whether a patient has a UTI, based on their symptoms and dipstick analysis’

	Univariate			Multivariate		
	OR	95% CI		OR	95% CI	
		Lower bound	Upper bound		Lower bound	Upper bound
Age (years)	0.99	0.97	1.01	***0.96***	0.94	0.99
Working experience (years)	***1.02***	1.00	1.04	***1.03***	1.01	1.06
Number of working days per week						
1	0.29	0.05	1.72	0.31	0.04	2.39
2	0.72	0.29	1.78	0.68	0.25	1.89
3	0.67	0.32	1.41	0.71	0.31	1.65
4	0.78	0.36	1.69	0.93	0.39	2.22
5	ref			ref		
Own UTI experience						
1–3 times	1.40	0.90	2.18	0.49	0.19	1.28
> 3 times in total, < 3 times per year	1.43	0.83	2.48	0.43	0.15	1.25
> 3 times per year	1.36	0.53	3.51	0.44	0.12	1.68
never	ref			ref		
Own UTI and AB experience	***1.82***	1.11	2.97	***3.31***	1.43	7.68

*AB antibiotic*,* CI confidence interval*,* GPA general practice assistant*,* OR odds ratio*,* UTI urinary tract infection*

#### UTI symptoms

We asked GPAs to choose two symptoms from a list of eight that they thought would most likely indicate a UTI in healthy, non-pregnant women. Most GPAs (79.9%) chose *dysuria* or *urinary frequency* (71.8%), with the combination of the two being chosen the most as well (55.6%) (Supplementary Table 2). Only 0.4% of the respondents correctly indicated the combination of *absence of vaginal complaints* and *dysuria* as most indicative of a UTI in healthy, non-pregnant women, as stated in the guidelines of the NHG [6].

#### High-risk groups for developing a complicated UTI

Almost a quarter of GPAs chose the correct high-risk groups for developing a complicated UTI, out of the 10 options presented (Supplementary Table 3). This was negatively correlated with GPAs’ age (OR = 0.96, 95%CI = 0.93–0.99) (Table 4). Patients with long-term urinary catheters and children below 12 years old were chosen the least frequently, 63.0% and 49.6%, respectively. Remarkably, 129 GPAs (27.0%) *slightly agreed* and 277 (57.9%) *strongly agreed* on the statement ‘*My knowledge about UTIs is sufficient to prescribe antibiotics to patients who are not at higher risk to develop a complicated UTI*,* without consulting a GP’*. Younger and more experienced GPAs were more likely to agree with this statement (OR = 0.97, 95%CI = 0.95-1.00 and OR = 1.06, 95%CI = 1.03–1.09, respectively) (Supplementary Table 4).

**Table 4:** Binary regression results for GPAs recognising the correct high-risk groups for developing a complicated UTI

	Univariate			Multivariate		
	OR	95% CI		OR	95% CI	
		Lower bound	Upper bound		Lower bound	Upper bound
Age (years)	***0.98***	0.96	1.00	***0.96***	0.93	0.99
Working experience (years)	1.00	0.98	1.02	1.02	0.99	1.06
Number of working days per week						
1	1.29	0.12	13.70	2.24	0.19	26.72
2	1.09	0.34	3.47	1.34	0.37	4.87
3	1.73	0.69	4.35	2.18	0.76	6.25
4	2.14	0.83	5.49	2.46	0.86	7.08
5	ref			ref		
Own UTI experience						
1–3 times	0.69	0.42	1.14	1.40	0.45	4.35
> 3 times in total, <3 times per year	0.97	0.53	1.77	1.97	0.56	6.94
> 3 times per year	0.99	0.36	2.78	2.19	0.48	10.02
never	ref			ref		
Own UTI and AB experience	0.95	0.52	1.72	1.35	0.51	3.60

*AB antibiotic*,* CI confidence interval*,* GPA general practice assistant*,* OR odds ratio*,* UTI urinary tract infection*

Bold are significant odd ratios

#### Diagnosis and treatment

Five true-false questions tested respondents’ general knowledge on the treatment and diagnosis of UTI. Among the respondents, 11.9% answered all questions correctly (Supplementary Table 5). However, 68% of respondents incorrectly agreed with the statement “In case of patients with a long-term bladder catheter, abdominal pain, a changed odour or consistency of the urine is a reason to perform urinary analysis”. Additionally, one-fourth (26.8%) of the GPAs incorrectly believed that a UTI can be diagnosed in case of a negative nitrite result but a positive leukocyte esterase and erythrocyte result, if a patient presents with non-UTI-specific complaints. Binary logistic regression did not reveal any variables significantly influencing GPAs knowledge (Table 5).

**Table 5:** Binary regression results for GPAs answering all true-false questions correctly

	Univariate			Multivariate		
	OR	95% CI		OR	95% CI	
		Lower bound	Upper bound		Lower bound	Upper bound
Age (years)	1.00	0.98	1.03	0.98	0.96	1.02
Working experience (years)	1.02	0.99	1.04	1.02	0.99	1.08
Number of working days per week						
1	1.60	0.15	17.38	2.19	0.17	28.36
2	1.15	0.33	3.97	1.37	0.34	5.57
3	0.94	0.34	2.58	0.97	0.30	3.17
4	0.62	0.21	1.87	0.59	0.17	2.08
5	ref			ref		
Own UTI experience						
1–3 times	1.48	0.72	3.04	0.66	0.09	5.00
> 3 times in total, < 3 times per year	1.22	0.51	2.96	0.48	0.06	4.16
> 3 times per year	1.00	0.20	4.85	0.41	0.03	5.12
never	ref			ref		
Own UTI and AB experience	0.50	0.19	1.30	0.34	0.06	2.14

*AB antibiotic*,* CI confidence interval*,* GPA general practice assistant*,* OR odds ratio*,* UTI urinary tract infection*

### GPAs’ attitude and practice

#### Diagnosis and treatment

On average, GPAs indicated to manage 4.8 out of 10 UTI-related cases completely independently. Half of GPA’s believed *Symptom relief* to be the most important reason for patients to consult a GP, followed by *obtaining antibiotics* (32.8%) (Supplementary Table 6). If a patient presents with symptoms that do not indicate a UTI but hands in urine anyway, most of the GPAs would *always* (44.8%) or *often* (24.9%) perform urinalysis, as a precaution. This was unaffected by GPAs’ characteristics (Supplemental Table 7). Additionally, 90.4% *never* ignored dipstick results when performed on urine from patients that do not suffer from UTI-related symptoms. When a UTI is diagnosed, three-fourth of the GPAs would *never* or *sometimes* (74.3%) advise a wait-and-see policy. Furthermore, over 50% of the respondents indicated a *positive urinary dipstick result* as the most important reason to prescribe an antibiotic (Supplementary Table 6).

#### Shared decision-making

Most GPAs (85.4%) indicated to apply SDM during their daily tasks regarding UTI-management. More than half of the GPAs (55.0%) *never* or only *sometimes* ask for patients’ expectations when they present with UTI-related symptoms. Remarkably, 74.9% considers *patients’ expectations* as the least important factor in deciding whether to prescribe antibiotics (Supplementary Table 6). Still, nearly three-fourths of the participants (74.7%) reported to *often* or *always* take patients’ opinion or wishes into account when providing advice.

Most of the respondents (30.5%) indicated ‘patients’ limited knowledge’ to be the most important barrier to applying SDM in practice, followed by time constraints. According to the participating GPAs facilitators of SDM are i.a. more available time, being able to inform patients more accurately and convincingly, more patient confidence in GPAs’ advice instead of only accepting advice given by GPs, and clear protocols or guidelines on SDM. Almost one-fourth (23.4%) desired training on how to apply SDM in practice.

#### Points for improvement in UTI-care according to GPAs

In an open-question GPAs were asked what their main point of improvement regarding UTI-care was. Decreasing the number of UTI-cases that is treated with antibiotics by more frequently applying a wait-and-see policy and giving non-antibiotic advice, was mentioned most frequently. Also, the need to adjust patients’ expectations to always receive an antibiotic treatment in case of a UTI, by informing them better about UTIs and its treatment options was mentioned regularly. GPAs indicated to desire training programs on when to advise a wait-and-see policy or prescribe antibiotics, and how to discuss this with a patient in a SDM-manner, the most.

## Discussion

### Summary of main findings

The results of this study comfirm that GPAs play a crucial role in UTI care in general practice, since they manage almost half of the UTI episodes independently. While over three quarters of GPAs recognized dysuria as the most important symptom for UTI, only one in four respondents selected the right risk factors of developing a complicated UTI. Moreover, nearly three-fourth of the GPAs would still perform urinalysis in absence of UTI symptoms. Although part of the current Dutch guideline, most GPAs do not regularly advice a wait-and-see policy in case of a UTI.

### Strengths and limitations

While we tried to ensure content validity by including GPAs and GPs from the start, we did not calculate the content validity index. Furthermore, it was difficult to determine construct validity of the survey, since no other methods of measuring GPA knowledge, attitudes, and practice were available for comparison. Another limitation of this study is the potential occurrence of selection bias due to our recruitment strategy, since GPAs specifically interested in UTIs may be more inclined to participate and may also be better informed about current guidelines [15]. Recruitment via social media might be especially prone to this. There is also the possibility of response bias, since respondents may have answered the questions in a socially desired way [16]. However, this might mean that their actual practice is more discordant with national guidelines than we found in our results. In addition, multiple-choice questions may not provide all possible answers, potentially contributing to response bias as well. Moreover, some survey questions could have been misinterpreted leading to inaccurate results.

The main strength of this study is its sample size of 478 participants. Secondly, the study population is a representative reflection of the actual population of GPAs in the Netherlands, since the average age years of profession in the study population are similar to the national average. The proportion of participating GPAs per province generally corresponds to the percentage of GP’s offices per province, except for overrepresented Limburg and underrepresented Noord-Holland [17]. Furthermore, to our knowledge, this is the first quantitative study examining GPAs’ knowledge, attitude, and practice in primary UTI-care in the Netherlands, which sheds an important light on how UTI-management in Dutch general practice can be improved.

### Comparison with existing literature

Most GPAs (~ 80%) indicated the presence of *dysuria* as most indicative of a UTI in non-pregnant, healthy women, followed by *urinary frequency*. A similar study performed among GPs found similar results. While the proportion of GPs that chose *dysuria* was larger at 90%, the spread among the symptoms was comparable. While both GPAs and GPs also look out for less specific symptoms, both groups know to look out for dysuria as the most important predictor of UTI [18–22]. The Dutch UTI guidelines also mention to look out for vaginal symptoms as a negative predictor for UTI. We intended to test if GPAs and GPs knew this by including *absence of vaginal symptoms* among the symptoms. In both studies 1% or fewer of respondents opted for this option. We hypothesise that this is not an accurate reflection of the respondents’ knowledge of the guidelines, but due to poor construction of the survey question. After all, it might not be intuitive to choose the option that indicates the lack of a symptom when asked to pick symptoms indicative of UTI.

In the same study, 65% of GPs indicated to not disregard the results of a urine dipstick if the patient’s symptoms are not indicative of a UTI [10]. Our results show that the result of the dipstick is of even greater importance to GPAs, since > 90% of GPAs would never disregard the dipstick result. This is especially concerning when considering GPAs high willingness to perform urinalysis, even in patients without UTI specific symptoms. Due to the urine dipsticks inadequate accuracy, GPAs high willingness to perform urinalysis and low willingness to disregard inappropriately acquired results will lead to significant overdiagnosis of UTI. Since GPAs indicated a positive dipstick result to be the most important reason to prescribe an antibiotic, this will lead to overtreatment with antibiotics as well [10, 23, 24]. Especially patients with asymptomatic bacteriuria will pose a problem, since they will test positive on the urine dipstick as well despite not needing to be treated in most cases [25–27]. The inappropriate use of antibiotics leads to increased antibiotic resistance as well as adverse events for the patient that could have been avoided. Furthermore, the misuse of diagnostics means that what is actually ailing the patient remains unknown.

GPAs’ age and working experience often significantly affect the respondents’ answers to the different questions. Strikingly, age and working experience often affect respondents’ answers in different directions. For example, younger GPAs answered were often more confident in their own abilities, while this was also the case for GPAs with more working experience. This seems paradoxical, since younger GPAs should have less working experience. However, we hypothesise that GPAs that recently finished their professional training with some years of working experience might be most familiar with the current guidelines. These GPAs will generally be young, which might explain the influence of age on the answers to the questions.

Most GPAs indicated to apply SDM during their daily tasks regarding UTI-management. It is therefore surprising that more than half of respondents never or only sometimes asks for patient preferences. Despite the added explanation of SDM in our survey GPAs might have the wrong idea of what SDM entails. Previous qualitative research has shown that SDM is rarely employed for UTI in Dutch general practice, especially in the case of incident cases [12]. It is therefore plausible that the low regard for patient expectations reflects daily practice more than the answer regarding SDM. Applying SDM could reduce the number of unnecessary antibiotic prescriptions, since 70% of surveyed women would be willing to accept a delayed antibiotic prescription [23, 28]. While the willingness to delay antibiotic prescriptions might be lower in practice, enquiring about the patient’s willingness during SDM may contribute to reducing antibiotic prescriptions. To this end, the NHG recently developed a ‘choice-card for women diagnosed with a UTI’ that succinctly outlines the 3 possible treatments options for UTIs to discuss with patients: wait-and-see policy, delayed- or immediate antibiotic prescriptions [29]. This tool could be a potential stimulator for GPAs to implement SDM in their daily tasks on UTI-care.

To keep primary care accessible in the future certain tasks may need to be delegated away from the GP to supporting personnel. The relative straightforwardness of uncomplicated UTIs make them an ideal indication to be officially delegated to GPAs, especially since GPAs seem to be managing most cases on their own anyway. In the Dutch healthcare system, GPs are allowed to delegate medical tasks to GPAs if the following conditions are met: GPAs need to be taught specific knowledge and skills about the task in question, there need to be clear protocols about the task, and GPs need to regularly check if the GPAs are skilled and if they follow the established protocols correctly. Therefore, GP practices could train their GPAs to manage uncomplicated UTI cases independently, so that GPs will have more time for the cases with a higher risk for a complicated course of disease. The population generally managed independently by GPAs is as of yet unknown, but this way GPAs limited knowledge on complicated UTIs would not be a limiting factor. As it stands, however, GPAs also seem to lack knowledge regarding uncomplicated cases and perhaps training at the practice only is not sufficient. GPAs are not required to follow additional education after graduation and therefore education directly aimed at GPAs is limited and variable per region. A centralised educational program could perhaps be more motivating than the GPs at the own practice that might have a smaller interest for UTIs. Furthermore, as shown by Gabbay et al., “mindlines” rather than guidelines seem to guide disease management in general practice [30, 31]. Mindlines are collectively reinforced, internalised tacit guidelines informed by brief reading of guidelines supplemented by interactions with opinion leaders, patients, and colleagues. Since GPAs obtain all updates on current guidelines from GPs in their practice, it is difficult to add to these established mindlines. To empower GPAs in their contribution, centralised continuing education might be prudent. This way both GPs and GPAs might follow the guidelines more closely. GPAs might feel more secure in denying patients urinalysis when it is not indicated and more secure in performing SDM. There has been little research on delegation of tasks to GPAs by GPs, and with the current and future challenges faced by GPs this might become an increasingly interesting avenue for research. This survey was a first exploration into the knowledge, practice, and attitudes of GPAs and subsequent qualitative research could shed further light on these aspects.

## Conclusions

The results of our study revealed that although almost all GPAs think their knowledge is sufficient to inform patients accurately about UTIs, this does not consistently hold true. By developing training programs specifically focusing on when urinalysis needs to be performed and why, GPAs might become more prudent when diagnosing UTI. Furthermore, GPAs should adjust their preconceived notions of patient preferences, since patients’ willingness to try non-antibiotic treatments is higher than they think. GPAs require more training in SDM and the time to apply it in order to make this possible.

## Supplementary Information

Supplementary Material 1.

## Data Availability

The survey and resulting data will be made available upon reasonable request. Interested parties can contact the corresponding author (Stefan Cox, [s.cox@maastrichtuniversity.nl](mailto: s.cox@maastrichtuniversity.nl)) for inquiry.

## Abbreviations

AB: Antibiotic
CI: Confidence interval
GP: General practitioner
GPA: General practice assistant
IP & AMR: Infection Prevention & Antimicrobial Resistance
NHG: Nederlands Huisartsen Genootschap (Dutch College of General Practitioners)
OR: Odds ratio
SD: Standard deviation
SDM: Shared decision making
UTI: Urinary tract infection
