# Supplementary material for: Knowledge, attitudes, and practice around urinary tract infections of general practice assistants in the Netherlands: a cross-sectional internet survey
Source: BMC Prim Care. 2025 Nov 3;26:338. doi: 10.1186/s12875-025-03025-3 (PMC12581395; doi:10.1186/s12875-025-03025-3)
Supplement: Supplementary file 1 — Supplementary Material 1. [file 12875_2025_3025_MOESM1_ESM.docx]

# Supplementary file 1

**Q1** Please indicate how often you perform the following actions associated with diagnosing cystitis:

|  | Never (1) | Sometimes (2) | Often (3) | Always (4) |
| --- | --- | --- | --- | --- |
| When a patient presents with symptoms possibly indicating a cystitis, I ask whether they could specify their symptoms (what has the patient been feeling exactly and for how long) (1) |  |  |  |  |
| When a patient presents with complaints possibly indicating a cystitis, I ask for the patients expectations (request for medical assistance) (2) |  |  |  |  |
| When a patient presents with complaints possibly indicating a cystitis, I ask for/examine the patient’s medical history (3) |  |  |  |  |
| I instruct patients to fill out the UTI-questionnaire available on thuisarts.nl when they hand in their urine (4) |  |  |  |  |
| When urine is handed in by patients that experience symptoms not indicating a cystitis, I still perform a urine dipstick test, just to be sure (5) |  |  |  |  |
| When urine is handed in by patients that experience symptoms not indicating a cystitis, I ignore the results of the urine dipstick test (6) |  |  |  |  |

**Q2** Please indicate to which degree you agree with the following statements regarding the diagnosis of cystitis:

|  | Completely disagree (1) | Somewhat disagree (2) | Neutral (3) | Somewhat agree (4) | Completely agree (5) |
| --- | --- | --- | --- | --- | --- |
| I have sufficient knowledge to estimate whether someone has a cystitis based on their symptoms and urine dipstick result (1) |  |  |  |  |  |
| Using the current urine dipstick I am able to determine the presence of a cystitis with sufficient certainty (2) |  |  |  |  |  |
| Using the current urine dipstick I am able to determine the absence of a cystitis with sufficient certainty (3) |  |  |  |  |  |
| I think the results of the urine dipstick are easy to interpret (4) |  |  |  |  |  |
| I need other diagnostic (urine) tests that the current urine dipstick (5) |  |  |  |  |  |
| Our practice has a clear protocol for cystitis available (6) |  |  |  |  |  |

|  |  |
| --- | --- |

**Q3** The combination of which 2 symptoms makes the diagnosis ‘cystitis’ the most likely for an otherwise healthy, non-pregnant woman?
 *Choose 2 symptoms from the following list*

- Painful urination (dysuria) (1)
- Lower abdominal pain (2)
- Having to urinate often in small amounts (frequent urination) (3)
- Malodorous or turbid urine (4)
- Urge to urinate on an empty bladder (5)
- Bloody urine (haematuria) (6)
- Fever or shivers (7)
- Absence of vaginal complaints (8)

**Q4** Please indicate how frequent you perform the following actions:

|  | Never (1) | Sometimes (2) | Often (3) | Always (4) |
| --- | --- | --- | --- | --- |
| Once I have diagnosed a cystitis, I advise the patient to drink enough water and to take pain medication, so that the symptoms will resolve spontaneously (wait-and-see) (1) |  |  |  |  |
| I consider the patient’s opinions/wishes when I give my advice (2) |  |  |  |  |
| Once I have diagnosed a cystitis, I prescribe an antibiotic without consulting a GP (GP only has to sign off) (3) |  |  |  |  |
| I refer patients with a cystitis to **www.thuisarts.nl** (4) |  |  |  |  |

**Q5** Please indicate to what degree you agree with the following statements regarding the treatment of cystitis:

|  | Completely disagree (1) | Somewhat disagree (2) | Neutral (3) | Somewhat agree (4) | Completely agree (5) |
| --- | --- | --- | --- | --- | --- |
| I am able to inform a patient correctly and completely about cystitis (1) |  |  |  |  |  |
| My knowledge on cystitis is sufficient enough to prescribe antibiotics to patient that do not belong to a risk group without consulting a GP (2) |  |  |  |  |  |
| I schedule a patient for consultation when I see that he or she has recurrent bladder infections (≥3 times per year) (3) |  |  |  |  |  |

**Q6** What proportion of patients who present with urinary complaints that may be consistent with a bladder infection do you, according to your estimate, treat without consulting a GP and therefore completely independently (from telephone call to giving advice or prescribing antibiotics)?

▼ I do not treat patients without consulting a GP (1) ... I treat all patients completely independently (11)

*Please indicate whether you think the following statements are correct or incorrect*

**Q7** A UTI can resolve spontaneously by drinking enough and possibly taking pain medication

- Correct (1)
- Incorrect (2)
- Do not know (3)

**Q8** When a patient has non-cystitis-specific complaints and the urine stick is negative for nitrite, the diagnosis of cystitis can be made based on a positive leukocyte and erythrocyte result

- Correct (1)
- Incorrect (2)
- Do not know (3)

**Q9** In case of fever or other signs of a kidney infection, a urine culture should be performed

- Correct (1)
- Incorrect (2)
- Do not know (3)

**Q10** In the case of patients with an indwelling catheter, abdominal pain, a change in urine odour or consistency (cloudy urine) is a reason to perform a urine test

- Correct (1)
- Incorrect (2)
- Do not know (3)

**Q11** When a patient is correctly treated for a cystitis with an antibiotic, antibiotic resistance cannot occur

- Correct (1)
- Incorrect (2)
- Do not know (3)

**Q12** Which group(s) has/have a greater risk of a complicated UTI?
 *Multiple answers possible*

- Children (1)
- Patients with kidney or urinary tract abnormalities (2)
- Diabetes mellitus patients (3)
- Patients with an indwelling catheter (4)
- Men (5)
- Pregnant women (6)
- Smokers (7)
- Overweight patients (8)
- Post-menopausal women (9)
- Patients with a history of UTI (10)

**Q13** What do you advise if a patient has a bladder infection?
 *Multiple answers possible*

- Empty the bladder completely (1)
- Do not delay urination when you have the urge to urinate (2)
- Consume cranberry tablets or drink (3)
- Take pain medication (4)
- Drink sufficiently (5)
- Take vitamin C (6)
- Avoid walking or sitting on a cold surface (7)
- Other, namely: (8)

**Q14** What factors are most important to you when deciding whether a patient should receive an antibiotic for a cystitis?

*Prioritize the options below by dragging them to the desired position. 1 represents the most important factor and 5 the least important.*

______ Patient symptoms (1)

______ Symptom duration (2)

______ Medical history (3)

______ Patient’s expectation/wish (4)

______ Positive urine dipstick test (5)

**Q15** If a new diagnostic test for urinary tract infections is designed, what would you consider to be the most important requirement??
 *Prioritize the options below by dragging them to the desired position. 1 represents the most important factor and 5 the least important.*

______ Optimise ease of use (1)

______ Limiting patient discomfort (2)

______ Limit time-to-result (3)

______ Maximizing the certainty with which the diagnosis can be made (4)

______ Limiting the costs (5)

**Q16** With what reason do you expect most patients contact a GP practice when they think they have a bladder infection??
 *Prioritize the options below by dragging them to the desired position. 1 represents the most important expectation and 5 the least important.*

______ Obtaining a diagnosis (1)

______ Obtaining reassurance (2)

______ Obtaining symptom relief (3)

______ Obtaining additional examination (4)

______ Obtaining an antibiotic prescription (5)

*Below are some questions about shared decision-making:*

*Shared decision-making is a process in which a healthcare provider (doctor, doctor's assistant, etc.) decides about diagnosis, treatment or (future) care together with the patient. For example, deciding together whether you are going to wait and see if the complaints resolve spontaneously or whether the patient starts taking an antibiotic. In addition, it can also be decided together whether urine is to be submitted and tested at all.*

**Q17** Do you apply shared decision making in your UTI management activities?

- Yes (1)
- No (2)
- Do not know (3)

**Q18** If anything, what most hinders you from engaging in shared decision-making?

- Insufficient knowledge among general practice assistants (1)
- Limited knowledge among patients (2)
- Insufficient experience/training (3)
- Time pressure (4)
- I am not hindered in engaging in shared decision-making (5)
- Other, namely: (6) __________________________________________________

**Q19** What would help you to apply shared decision-making (more often) in your daily activities?
 *Write down the aspect most important to you. If you are not hindered in performing shared decision-making, leave this question unanswered.*

________________________________________________________________

**Q20** Would you like (additional) training on shared decision-making??

- Yes (1)
- No (2)

**Q21** Which aspect of current care for urinary tract infections in general practice do you think needs to be improved?
 *Write down the aspect most important to you. If there are no points for improvement, leave this question unanswered.*

________________________________________________________________

**Q22** What is your age?

________________________________________________________________

**Q23** What is your gender?

- Man (1)
- Woman (2)
- Other (3)

**Q24** What is your current living situation?

- Living alone (1)
- Living together with a partner (2)
- Living together with partner and children (3)
- Other, namely: (4) __________________________________________________

**Q25** What is your current work situation?

- Working as a GPA (1)
- GPA in training (2)
- GPA, currently inactive (3)
- Other, namely: (4) __________________________________________________

**Q26** How many years of experience working as a GPA do you have?

________________________________________________________________

**Q27** On average, how many days a week do you work?

▼ 0 (1) ... 7 (8)

**Q28** In which region do you work most often currently?

▼ Limburg (1) ... Zuid-Holland (12)

**Q29** Did you ever experience a cystitis yourself?

- Yes, a few times (1-3 times total) (1)
- Yes, more often (>3 times total, but <3 times per year) (2)
- Yes, regularly (≥3 times per year) (3)
- No (4)
- Do not know (5)

**Q30** Have you ever used antibiotics yourself to treat a cystitis?

- Yes, how often? (1) __________________________________________________
- No (2)
- Not applicable (I have never had a cystitis) (3)
- Do not know (4)

**Q31** Do you need additional training on urinary tract infection care in general practice? If so, on what?

- Yes, specifically regarding: (1) __________________________________________________
- No (2)

**Q32** If you have any questions or comments about this questionnaire, or about urinary tract infections in general practice, please post them below:
 *If not, then you can leave the box empty.*

________________________________________________________________

**Supplementary Table 1** Ordinal regression results for the agreement of GPAs with the statement 'I am capable of providing a patient comprehensive and accurate information about UTIs'.

|  |  | **Univariate** | | | | **Multivariate** | | | |
| --- | --- | --- | --- | --- | --- | --- | --- | --- | --- |
|  |  | **OR** | **95% CI** | | | **OR** | **95% CI** | |  |
|  |  |  | **Lower bound** | **Upper bound** |  | | **Lower bound** | **Upper bound** |  |
| **Age (years)** |  | 1.00 | 0.89 | 1.21 | 1.00 | | 0.98 | 1.03 |  |
| **Working experience (years)** |  | ***1.03*** | 1.02 | 1.05 | ***1.04*** | | 1.01 | 1.07 |  |
| **Own UTI experience** | 1-3 times | ***1.56*** | 1.00 | 2.41 | 0.77 | | 0.29 | 2.07 |  |
|  | <3 times per year, >3 times in total | ***2.41*** | 1.37 | 4.22 | 1.31 | | 0.43 | 3.96 |  |
|  | >3 times per year | 2.63 | 0.95 | 7.26 | 1.64 | | 0.39 | 6.83 |  |
|  | Never | ref |  |  | ref | |  |  |  |
| **Number of working days per week** | 1 | 0.20 | 0.03 | 1.26 | 0.14 | | 0.02 | 1.11 |  |
|  | 2 | 0.42 | 0.17 | 1.03 | 0.39 | | 0.14 | 1.08 |  |
|  | 3 | 0.61 | 0.29 | 1.26 | 0.48 | | 0.20 | 1.11 |  |
|  | 4 | 0.98 | 0.45 | 2.12 | 0.96 | | 0.40 | 2.31 |  |
|  | 5 | ref |  |  | ref | |  |  |  |
| **Own UTI and AB experience** |  | ***1.71*** | 1.04 | 2.82 | 1.58 | | 0.66 | 3.77 |  |

*AB = antibiotic, CI = confidence interval, GPA = general practice assistant, OR = odds ratio, UTI = urinary tract infection*

**Supplementary Table 2** Top five most frequently chosen combinations of symptoms believed by GPAs to indicate a UTI (n=478).

| Symptom combination | Respondents (%) | Symptom | Respondents (%) |
| --- | --- | --- | --- |
| Dysuria + urinary frequency | 266 (55.6) | Dysuria | 382 (79.9) |
| Dysuria + urge without voiding | 59 (12.3) | Urinary frequency | 343 (71.8) |
| Urinary frequency + urge without voiding | 45 (9.4) | Urge without voiding | 119 (24.9) |
| Dysuria + malodorous or turbid urine | 31 (6.5) | Malodorous or turbid urine | 60 (12.6) |
| Uriney frequency + malodorous or turbid urine | 19 (4.0) | Lower abdominal pain | 25 (5.2) |
|  |  | Fever or shivers | 14 (2.9) |
|  |  | Hematuria | 10 (2.1) |
|  |  | Absence of vaginal complaints | 3 (0.6) |

*GPA = general practice assistant, UTI = urinary tract infection*

**Supplementary Table 3** Patients at higher risk of developing a complicated UTI according to GPAs (n=478).

| Risk factors |  | Respondents (%) |
| --- | --- | --- |
| Abnormalities of the urinary system | (correct) | 421 (88.1) |
| Diabetes mellitus | (correct) | 417 (87.2) |
| Pregnancy | (correct) | 393 (82.2) |
| Male gender | (correct) | 359 (75.1) |
| Long-term urinary catheters | (correct) | 301 (63.0) |
| Age <12 years | (correct) | 237 (49.6) |
| Post-menopausal women |  | 55 (11.5) |
| Medical history of a UTI |  | 54 (11.3) |
| Smoking |  | 22 (4.6) |
| Obesity |  | 18 (3.8) |
|  |  |  |
| All groups at higher risk correctly indicated |  | 119 (24.9) |

*UTI = urinary tract infection, GPA = general practice assistant.*

**Supplementary Table 4** Ordinal regression results for the agreement of GPAs with the statement ‘My knowledge about UTIs is sufficient to prescribe antibiotics to patients who are not at higher risk to develop a complicated UTI, without consulting a GP’.

|  |  | **Univariate** | | | **Multivariate** | | |
| --- | --- | --- | --- | --- | --- | --- | --- |
|  |  | **OR** | **95% CI** | | **OR** | **95% CI** | |
|  |  |  | **Lower bound** | **Upper bound** |  | **Lower bound** | **Upper bound** |
| **Age (years)** | | 1.01 | 0.99 | 1.02 | ***0.97*** | 0.95 | 1.00 |
| **Working experience (years)** | | ***1.04*** | 1.02 | 1.06 | ***1.06*** | 1.03 | 1.09 |
| **Number of working days per week** | 1 | 0.63 | 0.11 | 3.42 | 0.41 | 0.06 | 2.80 |
|  | 2 | 1.66 | 0.73 | 3.80 | 1.59 | 0.63 | 4.00 |
|  | 3 | 1.57 | 0.82 | 3.00 | 1.53 | 0.73 | 3.19 |
|  | 4 | 1.83 | 0.92 | 3.61 | 1.86 | 0.87 | 3.99 |
|  | 5 | ref |  |  | ref |  |  |
| **Own UTI experience** | 1-3 times | 1.16 | 0.75 | 1.78 | 0.50 | 0.20 | 1.27 |
|  | >3 times in total, <3 times per year | 1.19 | 0.70 | 2.02 | 0.47 | 0.16 | 1.34 |
|  | >3 times per year | 1.54 | 0.60 | 3.97 | 0.75 | 0.20 | 2.85 |
|  | never | ref |  |  | ref |  |  |
| **Own UTI and AB experience** | | 1.45 | 0.90 | 2.35 | 2.09 | 0.92 | 4.74 |

*AB = antibiotic, CI = confidence interval, GP = general practitioner, GPA = general practice assistant, OR = odds ratio, UTI = urinary tract infection*

**Supplementary Table 5** GPAs’ knowledge on diagnosing and treating UTIs (n=478).

| Statement | Respondents (%) |
| --- | --- |
| *A cystitis can resolve by increased fluid intake and using painkillers* | |
| True (correct) | 451 (94.4) |
| False | 24 (5.0) |
| Do not know | 3 (0.6) |
| *If a patient presents with non-cystitis-specific complaints and the dipstick analysis on nitrite is negative, a cystitis can be diagnosed based on a positive leukocyte esterase and erythrocyte result of the dipstick* | |
| True | 128 (26.8) |
| False (correct) | 331 (69.2) |
| Do not know | 19 (4.0) |
| *In case of a fever or other signs of tissue invasion, urine culture needs to be performed* | |
| True (correct) 378 (79.1) | |
| False 63 (13.2) | |
| Do not know 37 (7.7) | |
| *In case of patients with a long-term bladder catheter, abdominal pain, a changed odour, or consistency of the urine is a reason to perform urinary analysis* | |
| True 325 (68.0) | |
| False (correct) 119 (24.9) | |
| Do not know 34 (7.1) | |
| *When a patient is correctly treated for a cystitis using antibiotics, antibiotic resistance cannot occur* | |
| True 14 (2.9) | |
| False (correct) 418 (87.4) | |
| Do not know 46 (9.6) | |
|  | |
| All statements correctly answered 57 (11.9) | |

*GPA = general practice assistant, UTI = urinary tract infection*

**Supplementary Table 6** Most and least important features of new diagnostic urinary tests, considerations before prescribing an antibiotic, and patients’ reasons for consultation according to GPAs (n=478).

| Most important (%) |  | Least important (%) |
| --- | --- | --- |
|  | **Test feature** |  |
| 72.0 | Diagnostic accuracy | 1.3 |
| 10.0 | Time-to-result | 13.6 |
| 9.6 | Ease-to-use | 12.8 |
| 6.5 | Patient discomfort | 20.7 |
| 1.9 | Costs | 51.7 |
|  | **Consideration before prescribing an antibiotic** |  |
| 56.7 | Positive urinary dipstick test | 5.2 |
| 24.5 | Patient’s symptoms | 2.1 |
| 11.5 | Patient’s medical history | 5.4 |
| 4.4 | Duration of patient’s symptoms | 12.3 |
| 2.9 | Patient’s expectation | 74.9 |
|  | **Patients’ reasons for consultation** |  |
| 50.0 | Symptom relief | 0.6 |
| 32.8 | Obtaining an antibiotic | 3.1 |
| 14.9 | Diagnosis | 4.0 |
| 1.5 | Additional examination | 59.2 |
| 0.8 | Reassurance | 33.1 |

*GPA = general practice assistant*

**Supplementary Tabel 7** Ordinal regression results of GPAs’ agreement with the statement ‘If urine is handed in by a patient with symptoms that are not indicative of a UTI, then I will still perform urinalysis as a precaution’.

|  |  | **Univariate** | | | **Multivariate** | | |
| --- | --- | --- | --- | --- | --- | --- | --- |
|  |  | **OR** | **95% CI** | | **OR** | **95% CI** | |
|  |  |  | **Lower bound** | **Upper bound** |  | **Lower bound** | **Upper bound** |
| **Age (years)** | | 1.00 | 0.99 | 1.02 | 1.01 | 0.99 | 1.03 |
| **Working experience (years)** | | 1.01 | 0.99 | 1.02 | 1.01 | 0.99 | 1.03 |
| **Number of working days per week** | 1 | 2.04 | 0.33 | 12.72 | 1.11 | 0.15 | 8.19 |
|  | 2 | 1.14 | 0.51 | 2.54 | 1.06 | 0.44 | 2.59 |
|  | 3 | 0.99 | 0.52 | 1.88 | 0.82 | 0.40 | 1.69 |
|  | 4 | 1.34 | 0.69 | 2.62 | 1.28 | 0.61 | 2.69 |
|  | 5 | Ref |  |  | Ref |  |  |
| **Own UTI experience** | 1-3 times | 0.78 | 0.51 | 1.17 | 0.50 | 0.20 | 1.25 |
|  | >3 times in total, <3 times per year | 0.97 | 0.59 | 1.62 | 0.61 | 0.22 | 1.69 |
|  | >3 times per year | 1.22 | 0.50 | 2.94 | 0.81 | 0.23 | 2.84 |
|  | never | Ref |  |  | Ref |  |  |
| **Own UTI and AB experience** | | 0.90 | 0.56 | 1.44 | 1.29 | 0.58 | 2.86 |

*AB = antibiotic, CI = confidence interval, GP = general practitioner, GPA = general practice assistant, OR = odds ratio, UTI = urinary tract infection*
